# Colorectal Cancer (CRC) treatment and associated costs in the public sector compared to the private sector in Johannesburg, South Africa

**DOI:** 10.1186/s12913-020-05112-w

**Published:** 2020-04-07

**Authors:** Candice-lee Herbst, Jacqueline K. Miot, Shirra L. Moch, Paul Ruff

**Affiliations:** 1 Division of Pharmacology, Department of Pharmacy and Pharmacology, Faculty of Health Sciences, University of the Witwatersrand, 7 York Road, Parktown, Johannesburg, 2193 South Africa; 2 Health Economics and Epidemiology Research Office, Department of Internal Medicine, Faculty of Health Sciences, University of Witwatersrand, 39 Empire Road, Parktown, Johannesburg, 2193 South Africa; 3 Centre for Health Science Education, Faculty of Health Sciences, University of the Witwatersrand, 7 York Road, Parktown, Johannesburg, 2193 South Africa; 4 Division of Medical Oncology, Faculty of Health Sciences, University of the Witwatersrand, 7 York Road, Parktown, Johannesburg, 2193 South Africa; 5 University of Witwatersrand / Medical Research Council Common Epithelial Cancers Research Centre (WITS/MRC CECRC), 7 York Road, Parktown, Johannesburg, 2193 South Africa

**Keywords:** Colorectal Cancer, Chemotherapy, Resource Utilization, Costs, UMIC

## Abstract

**Background:**

South Africa’s divided healthcare system is believed to be inequitable as the population serviced by each sector and the treatment received differs while annual healthcare expenditure is similar. The appropriateness of treatment received and in particular the cost of the same treatment between the sectors remains debatable and raises concerns around equitable healthcare. Colorectal cancer places considerable pressure on the funders, yet treatment utilization data and the associated costs of non-communicable diseases, in particular colorectal cancer, are limited for South Africa. Resources need to be appropriately managed while ensuring equitable healthcare is provided regardless of where the patient is able to receive their treatment. Therefore the aim of this study was to determine the cost of colorectal cancer treatment in a privately insured patient population in order to compare the costs and utilization to a previously published public sector patient cohort.

**Methods:**

Private sector costs were determined using de-identified claim-based data for all newly diagnosed CRC patients between 2012 and 2014. The costs obtained from this patient cohort were compared to previously published public sector data for the same period. The costs compared were costs incurred by the relevant sector funder and didn’t include out-of-pocket costs.

**Results:**

The comparison shows private sector patients gain access to more of the approved regimens (12 vs. 4) but the same regimens are more costly, for example CAPOX costs approximately €150 more per cycle. The cost difference between 5FU and capecitabine monotherapy is less than €30 per cycle however, irinotecan is cheaper in comparison to oxaliplatin in the private sector (FOLFOX approx. €500 vs. FOLFIRI aprox. €460). Administrative costs account for up to 45% of total costs compared to the previously published data of these costs totaling < 15% of the full treatment cost in South Africa’s public healthcare system.

**Conclusion:**

This comparison highlights the disparities between sectors while illustrating the need for further research to improve resource management to attain equitable healthcare.

## Background

Currently the South African healthcare system is divided into two healthcare sectors, namely public and private. While the majority of the South African population makes use of public healthcare (85%), only 15% subscribe to private medical insurance i.e. medical aid schemes, which must provide a prescribed minimum benefits package (PMB), similar to the care received in the public health care sector [1, 2]. Conversely the public healthcare system is funded by the yearly national income taxation collection and the resource allocation is overseen by the National Department of Health (NDoH) via the individual Provincial Health Departments [2]. Part of the resource allocation includes medicine selection and access through the Essential Drugs Program (EDP), comprising of the Essential Medicines List (EML) and Standard Treatment Guidelines (STGs). These are used as a guideline for the PMBs as set out by the Medical Schemes Act [3, 4].

Therefore the private healthcare sector is aimed at middle- and high-income earners to better cover their healthcare needs through increased access to medicines and healthcare professionals within the country [5]. Although medical services and medicines are covered by the medical insurance schemes, co-payments are frequently paid by the beneficiaries [1]. In addition, medicine selection is based on individual scheme formularies and benefit designs with regulated medicine pricing implemented by the NDoH to ensure cost transparency within the sector [6]. Although pricing and annual price adjustments ensure transparency, they do not govern the initial price of medicines as set out internationally by pharmaceutical companies. Cost differences are therefore common within the private sector per medicine class for a disease area, in particular cancer [7]. Overall total expenditures, incurred for the two funders remain similar between sectors despite the difference in the size of the population benefiting [1, 2]. This substantiates the belief that the South African healthcare system is inequitable especially for diseases where less attention is paid such as cancer.

A competitive bidding process occurs in the public healthcare sector allowing the best possible price to be obtained for medicines prescribed [8, 9]. Although this lowers the cost per medicine class; the range of choice between individual medicines for a class isn’t provided as is for the private healthcare sector.

Despite South Africa being classified as an Upper-Middle Income Country (UMIC) according to the 2016 World Bank Statistics the current available treatment for colorectal cancer (CRC) in South Africa differs between the two healthcare sectors (Table 1) [10, 11]. Additionally, private healthcare sector patients have access to many of the medicines available in High Income Countries (HICs) [12] such as the USA and EU [13–19]. Furthermore this difference not only influences the number of chemotherapy treatment regimens oncologists are able to prescribe in each sector but also the costs associated with CRC treatment. While practices including clinical treatment pathway implementation have been employed to curb the rising cost of cancer treatment in HICs, limited published information indicates that such clinical treatment pathways are not adequately in use in either healthcare sector within South Africa. EMLs and formularies do however direct medicine prescriptions in the two healthcare sectors (Table 1) [20–23].

**Table 1:** Chemotherapy medicine availability in South Africa per healthcare sector.

Healthcare Sector	Guideline chemotherapy selection	Medicines available
**Public**	Department of Health Master Procurement Catalogue (EML and STG)	5-FU (+LV), Capecitabine, Oxaliplatin, ^a^Irinotecan
**Private**	Various medical aid scheme formularies for the South African Health Products Regulatory Authority (previously Medicines Control Council) approved medicines	5-FU (+LV), Capecitabine, Oxaliplatin, Irinotecan, Bevacizumab, Cetuximab, ^b^Panitumumab, ^c^Aflibercept, ^b^Regorafenib

^a^ Subsequent to this study has been approved for use in the public sector. Medicines underlined are available in both healthcare sectors.

^b^Subsequent to this study Panitumumab and Regorafenib have been approved for use by SAHPRA - South African Health Products Regulatory Authority – but was previously available through a Section 21named patient use application.

^c^Not yet registered for use in South Africa but is available through Section 21 named patient application.

Recent research on a similar database to the one used in this study focuses on surgical procedures and outcomes for CRC in a privately insured patient cohort. While valuable information is obtained from this research it does not address issues around cost and concerns only one of South Africa’s health sectors [24]. Apart from this analysis, published literature regarding the costs in the private healthcare sector and differences between the sectors associated with receiving CRC chemotherapy is lacking. The determinations of these costs are an important and much-needed contribution as South Africa moves towards the implementation of Universal Health Coverage.

Thus the aim of this study was to compare a previously published South African public healthcare sector patient cohort’s medicine utilisation and the associated costs by the same authors (Herbst et al, 2018) to a private South African medical aid scheme’s claims (costs of chemotherapy submitted for payment) data for the same period.

## Methods

### Patient cohort database

The cohort inclusion criteria such as “newly diagnosed”, outpatient treatment setting and type of cancer treatment along with study period were based on a previously published cohort study performed for South Africa’s public healthcare sector thus allowing a comparison of costs between the sectors [25].

Three de-identified claim-based data sets were obtained from a private medical scheme and manually sorted to include all newly diagnosed CRC patients between 2012 and 2014. The claims data allowed for at least 12 months of follow-up data therefore costs up to the end of 2015 was requested. Only chemotherapy and related medicine treatment was included. The data sets received were named as follows, for the purpose of this study: *A1 – medical claims for chemotherapy and related medicine*, *A2 – non-medical claims* i.e. *administrative costs for outpatient services* and *A3 – Demographic and disease-related data.* Patients were excluded if diagnosis was prior to 2012 or after 2014, if no demographic data or non-medical data was received for any patients included in data set A1.

**Fig. 1:**
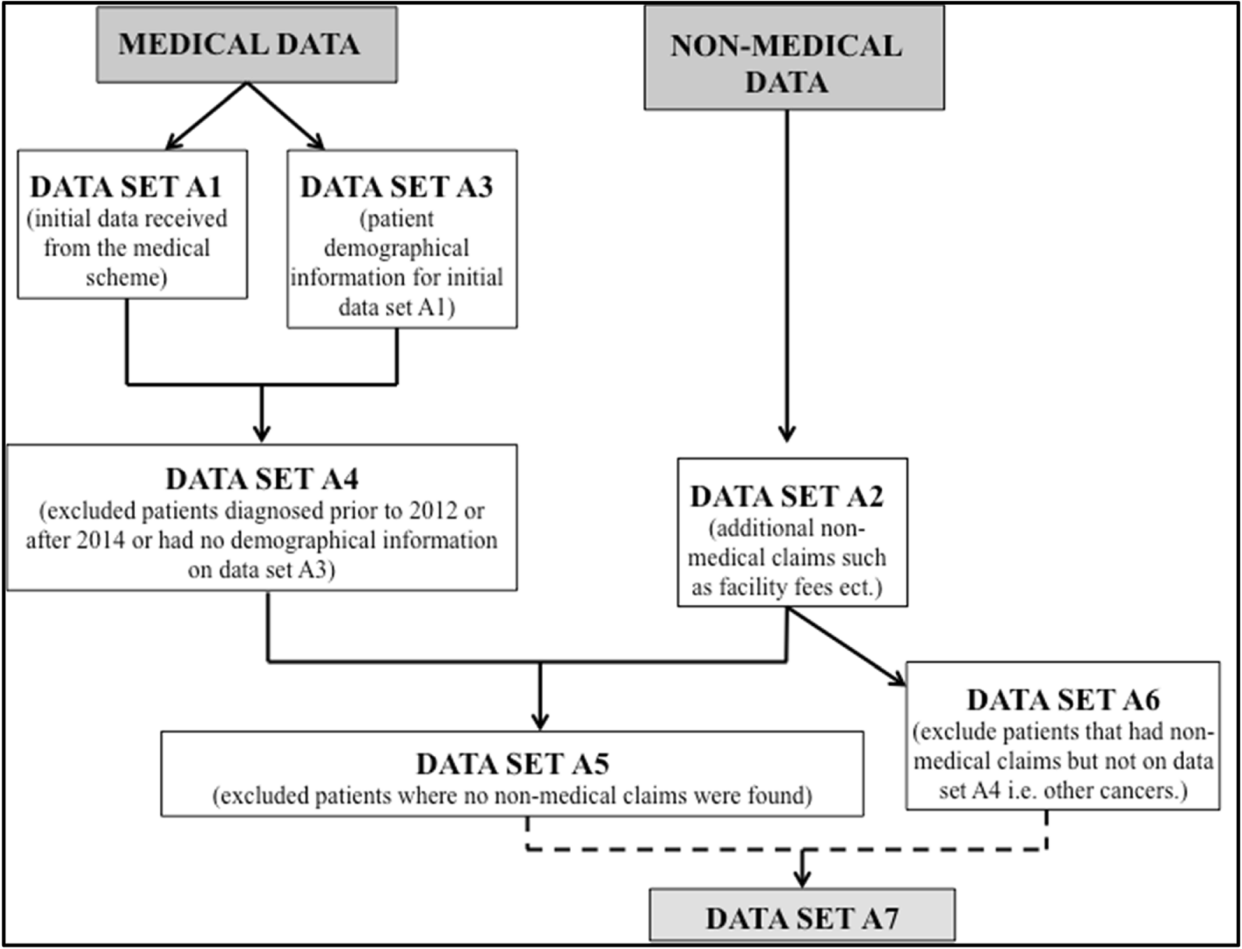
Flow diagram showing process of obtaining the final patient cohort included in the study.

All patient identifiers from each data set were coded and only known to the researchers for the duration of the study. The final complete data set comprised of two smaller data sets (Fig. 1: Flow diagram showing process of obtaining the final patient cohort included in the study).

### Patient cohort demographics and treatment pathways

Demographic data included age, gender, diagnosis and surgery. Patient diagnosis was simplified into early CRC (no evidence of metastasis found) [26] and late CRC (evidence of metastasis found) [27]. This initial diagnosis was established as per the data received from the medical scheme but altered to late CRC if subsequent evaluation from the treatment pathway indicated metastasis.

The per patient treatment pathways were manually derived from the final merged data set which was remodeled to include additional classification such as Anatomical Therapeutic Chemical (ATC) [28] for medicines, allowing each claim to be sorted into groups including administration medicine, chemotherapy, diagnostic/radiation medicine, pain management, supportive medicine or secondary supportive medicine. A two-dimensional pivot table was constructed in Excel for Mac (2011) to summarise each patient’s treatment and subsequently develop each patient’s treatment pathway according to sequential claim dates. Criteria applied in order to obtain the final treatment pathways and diagnoses per patient are seen in Table 2.

**Table 2:** Criteria applied to obtain final patient treatment pathways and diagnosis.

1. Treatment lines were determined by chemotherapy medicines grouped together if claimed over a single 3-month period.	
2. Diagnosis was finalised based on the data captured and classification made by the medical scheme and was changed to late CRC if a biological medicine was used in either 1st or 2nd line treatment or more than two lines of therapy were followed by a biological medicine.	
3. Each treatment line was colour-coded within the pathway for each patient and the treatment criteria were applied to finalize the number of treatment lines. A change in treatment line occurred if: • Oxaliplatin was switched to irinotecan or vice versa. • A biological medicine was included or changed to another biological medicine. No change in treatment line occurred if: • A medicine was not prescribed for a certain number of cycles. • 5-FU was switched to capecitabine or vice versa. • An oxaliplatin/irinotecan-containing regimen was changed to 5-FU/capecitabine monotherapy.	

Patient cohort demographics were analyzed which, included patient numbers per diagnosis as well as the mean, median and range of the age for each diagnostic sub-group. The number of patients that underwent surgery was also calculated.

### Per patient treatment costs

Using two-dimensional pivot tables all medical (medical claims for chemotherapy and related medicines) and non-medical (administrative costs for outpatient services) costs per patient, claimed through the private medical scheme, were collated. Claims data up to the end of 2015 was used to include at least 12 months of follow-up for patients enrolled in late 2014.

All claimed costs were adjusted to the last claimed cost in 2014 for each respective medicine or non-medical description in order to allow for comparisons to the public sector cohort results which, only published 2014 costing data [25]. All costs were converted to Euro’s using the average annual exchange rate of 1€ = ZAR14.40 (September 2018) [29].

If quantities claimed didn’t match the cost claimed, the quantities were adjusted to reflect the claimed costs. Medicines where no claim could be found for 2014 were adjusted to the August 2014 private sector medicines price database (http://www.mpr.gov.za/PublishedDocuments.aspx). However in instances where 2014 price was unavailable, the final adjusted price was calculated using the annual medicine increases [30–32]. Medicines obtained via Section 21 “named patient” approval and claims classified as “ethical nonspecific” (e.g. haemodialysis concentrate) were adjusted by the annual average Consumer Price Index (CPI) increase for 2014 as per the Inflation.eu website (https://www.inflation.eu) [33]. Using adjusted cost data all average costs per patient were calculated.

The total cost per cycle for each regimen observed in the cohort’s treatment pathways was filtered by CRC stage and the average claimed cost per medicine was determined so as to calculate the average cost per regimen (formulae in Supplement). The average cost per cycle for each regimen was determined using the treatment pathways developed in the cohort (formulae in Supplement). The average chemotherapy doses for each chemotherapy medicine were calculated to allow dosage comparisons based on the average cost per medicine as well as the cost per vial or tablets for the medicine using the lowest cost generic. This cost was selected to allow comparison between this cohort and the published public sector cohort data [25].

The non-medical costs were obtained by calculating the average administrative cost per regimen. The administrative costs included a global fee (fee charged for the management and services delivered during the treatment day) and a facility fee. For simplification, the global and facility fees were averaged for an oral and intravenous regimen. The costs per cycle and the total adjusted costs were calculated based on the average number of cycles per regimen from the medical data. It must be noted that administrative costs are independent of patient diagnosis but dependent on the chemotherapy administration. Consultation fees were excluded, as various medical specialties including medical oncologists, radiation oncologists and general practitioners submit differing claims but a consultation fee claim for every chemotherapy cycle was noted for every patient. Lastly, the total average cost per treatment regimen was calculated (formulae in Supplement).

### Comparison of cohort to previously published public sector data [25]

The demographics and average costs calculated for our cohort was compared to previously published public sector research, conducted by the same authors of this study, in order to establish if differences in CRC treatment, cost and access occurs [25]. The comparison was from the funder’s perspective i.e. the Medical Scheme (private sector) and Government (public sector).

Descriptive statistics was used to obtain the averages, means, medians and range for the data. Inferential statistics were not used in this costing study.

## Results

### Patient cohort demographics and treatment pathways

The private sector patient cohort comprised of 729 males (56%) and 567 females (44%) with a mean age, regardless of CRC stage, of 63 years (range 23–91 years). More patients were diagnosed, according to final classification, with early CRC (65%) vs. late CRC (35%). 84% of the cohort underwent primary surgery (~ 67% early CRC diagnosis). Left-sided vs. right-sided CRC classification data was unavailable at the time therefore analysis based on the origin of the cancer could not be performed. Based on the criteria used to determine a change in chemotherapy treatment line for each patient’s treatment pathway, up to 6 lines of chemotherapy treatment (including adjuvant chemotherapy) were found although not many patients received more than 2 lines of chemotherapy (approx. 7%) regardless of stage. According to the initial treatment regimen patients received, more early diagnosed CRC patients started on a capecitabine-containing regimen as opposed to late stage diagnosed CRC patients receiving a 5-FU containing regimen (~ 60% vs. 40%).

### Per patient treatment costs

**Fig. 2:**
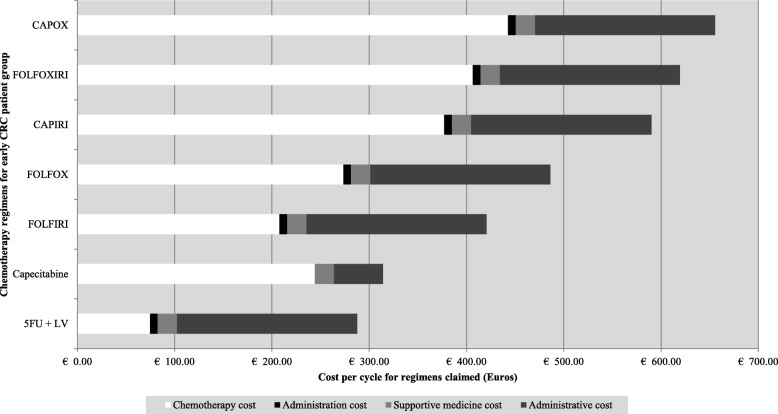
Early CRC regimen’s cycle cost for each claimed component as per the constructed treatment pathways.

**Fig. 3:**
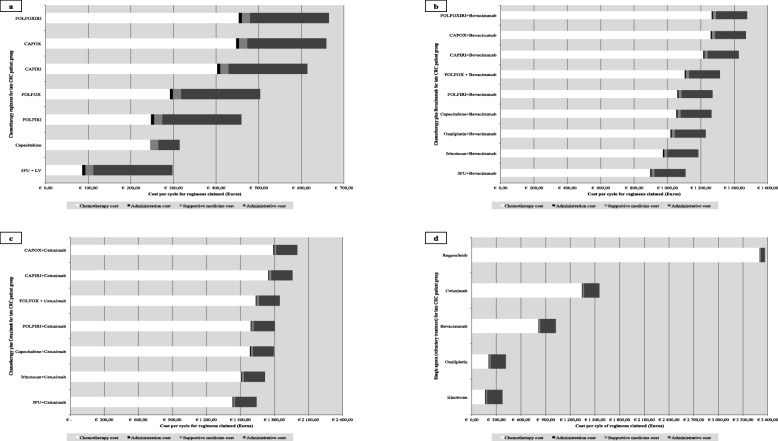
Late CRC regimen’s cycle cost for each claimed component as per the treatment pathways constructed - *(****a****– Chemotherapy alone;****b****– Chemotherapy plus Bevacizumab;****c****– Chemotherapy plus Cetuximab;****d****– Single agents for refractory patients).*

The largest cost component for the observed regimens, for either subgroup, was the cost of the chemotherapy, particularly regimens comprised of multiple chemotherapy agents. In addition administrative fees have a meaningful contribution to the overall cost per cycle (Fig. 2 and Fig. 3). The most expensive regimens per subgroup was found to differ, one such example was the use of FOLFOX + capecitabine for early CRC, which is unconventional and increases treatment costs, as the choice of regimen should either be FOLFOX or CAPOX.

Regorafenib was the most expensive regimen for the late CRC subgroup although this was only used in multi-refractory patients. Bevacizumab was cheaper than cetuximab, approx. €1000 vs. €1500 respectively. This is due to the difference in cost of the two monoclonal antibodies (Fig. 2: Early CRC regimen’s cycle cost for each claimed component as per the constructed treatment pathways and Fig. 3: Late CRC regimen’s cycle cost for each claimed component as per the treatment pathways constructed - *(A – chemotherapy alone; B – Chemotherapy plus Bevacizumab; C – Chemotherapy plus Cetuximab; D – Single agents for refractory patients)*). Unexpected results include the similar cost per cycle between 5-FU and capecitabine regimens for both the early and late subgroups, approx. €290 vs. €310 and €300 vs. €310 respectively. Cost per cycle for irinotecan monotherapy was cheaper than oxaliplatin monotherapy despite the increased cost of administration for irinotecan-containing regimens, for either subgroup, approx. €350 vs. €410 and €370 vs. €420 respectively.

### Comparison of cohort to previously published public sector data

The comparison between this study cohort and the previously published study highlights more differences than similarities. Apart from a similar gender split within the two cohorts (56% males: 44% females in this study vs. 55% males: 45% females), other demographic data such as age differ considerably (63 yrs. in this study vs. 57 yrs. in the public sector cohort). The stage at which patients are diagnosed in the private sector cohort is earlier than for the public sector cohort (35% vs 63%) and contributes to patients receiving more lines of treatment and therefore higher total treatment costs were observed.

## Discussion

The patient cohort included in this study is likely to be fairly representative of the private healthcare sector within South Africa, as the medical aid scheme population comprises one of the largest in the country. In comparison the public sector cohort was from only one public sector facility, albeit one servicing a large area within Johannesburg, the largest city within the country [25].

In comparison to the published public sector patient cohort [25]*,* the gender-proportions of the private sector cohort was similar. This trend follows the risk data seen in SEER (Surveillance, Epidemiology, and End Results Program) statistics [34]. Interestingly slightly more males than females are diagnosed within South Africa despite CRC being a non-gender specific disease [35].

Additionally, the number of females affected is lower in our cohort and could be as a result of their socioeconomic status, which influences differences in lifestyle. A prospective study conducted in Denmark found patients who adhere to health recommendations reduce their risk considerably [36]. The average diagnosis age for the public sector cohort was younger than our private sector cohort but the private sector patient cohort following similar global trends [37–39].

The stage at which the patients were diagnosed yields an interesting comparison. The number of patients with late CRC is greater in the previously studied public sector cohort [25]. Initially it was expected that due to the increased number of patients in our private sector cohort there may be more patients diagnosed with late CRC but when taking into account the socioeconomic status of the patients, healthcare resources and the asymptomatic timespan of the cancer, it is not unexpected to find more late presenting CRC patients in the public sector.

Assessing the number of treatment lines between the two patient cohorts illustrates the difference in access to treatment. As expected, a higher percentage of the metastatic subgroup received at least one line of chemotherapy in comparison to the non-metastatic subgroup (99% vs. 89%). More patients in the private sector cohort received 2nd (13% vs. 19%) and 3rd line treatments (0% vs. 5%), this is largely due to the absence of 3rd line treatments in South Africa’s public sector (Table 1) due to the limited number of available medicines in the public sector.

Reasons for the use of unconventional chemotherapy in the private sector cannot be ascertained from the data however it is suspected to be either off-label use or indicate the presence of a secondary cancer that was not captured in the claims database. Apart from the clinical inappropriateness, this adds an unnecessary contribution to each patient’s total cost of treatment. One such example was the recorded use of carboplatin + paclitaxel which increased the total cost by approximately €1600 (data not shown).

Comparing the treatment pathways developed in this study to international guidelines shows many treatments available elsewhere globally were available to private sector patients in South Africa. Standard therapies including bevacizumab and cetuximab were thus available to these patients unlike patients accessing public healthcare [13–15, 18, 19, 22, 40–44]. This gives some indication that chemotherapy treatment for CRC in South Africa does follow international trends. At the time of the study there was an absence of medicines such as aflibercept and panitumumab, although aflibercept was available via a named-patient regulatory approval process. Regorafenib was prescribed for a few patients, as it was also available on a named-patient basis at the time of the study, and will most likely be prescribed further since recent local regulatory approval, although availability will also be dependent on funding for reimbursement.

Looking at the first line treatments received in our patient cohort, capecitabine-containing regimens are favoured for early CRC patients (approx. 60%) whereas late CRC treatment pathways indicate a higher use of 5-FU-containing regimens (approx. 64%) even though capecitabine has proven non-inferiority to 5-FU for any stage of CRC [45]. It was unexpected to see a greater use of 5-FU for late CRC disease in the private sector but the majority of the regimens contain additional intravenous medicines thus it may be preference to receive treatment all at once. Many patients diagnosed with late CRC disease in our cohort have access to newer biological agents that can only be administered intravenously and may further contribute a preference for 5-FU when used in combination with conventional regimens. Studies have indicated patient preference for capecitabine due to less toxicity and ease of administration thus this does raise an important issue in the private sector as to what the drivers are for choice of treatment [46, 47].

In the previously published study, costs associated with late CRC treatment were higher than early CRC treatment [25] and the expectation was that our study would replicate this trend; however this proved incorrect, with the average cost per cycle being similar between the stages for the same regimens. This is essentially due to similar dosages, which was as a result of the assumption that the claimed vials were the prescribed doses. However in clinical practice the dosage may be lower due to the occurrence of vial wastage in order to accommodate BMI (body mass index) or body weight dosing. Wastage cost can’t be calculated from a claims database however these factors should be considered as published data by Bach and colleagues (2016) found that single-dose vials can lead to overspending as the vial sizes don’t match the prescribed doses for many medicines. In addition to this, vial sharing may also occur in larger practices [48, 49]. While vial sharing limits the wastage of viable medicines and potentially curbs overall treatment costs, the funder is billed for the entire vial thus clinical practice data of dose and cost doesn’t necessarily match. Vial sharing is one method suggested to curb costs however it is not recommended for all intravenous medicines [48, 49].

This observation is seen between both CRC subgroups for conventional regimens such as FOLFOX and FOLFIRI, where the difference is less than €50 per cycle (FOLFOX: approx. €480 vs. €500 and FOLFIRI: approx. €420 vs. €460) as seen in Fig. 2: Early CRC regimen’s cycle cost for each claimed component as per the constructed treatment pathways and Fig. 3: Late CRC regimen’s Cycle cost for each claimed component as per the treatment pathways constructed - *(A – chemotherapy alone; B – Chemotherapy plus Bevacizumab; C – Chemotherapy plus Cetuximab; D – Single agents for refractory patients)*. When comparing the costs for these regimens to the previously published study a few differences are noted [25]. Firstly, there is no possible comparison between the two early CRC subgroups as patients in the South African public sector cohort did not have access to regimens such as FOLFOX or FOLFIRI but comparing a non-inferior regimen CAPOX for either stage shows that the cost per cycle is much higher in our private sector patient cohort. The CAPOX regimen is in the range of €300 to €450 per cycle in the public sector [25] but costs more than €600 in our cohort. This illustrates that the cost to the funder, is higher in the private sector. Similarly to the findings from the previous published public sector cohort, irinotecan-containing regimens – FOLFIRI and CAPIRI cost less per cycle than regimens containing oxaliplatin. On average these regimens are €55 cheaper depending on the fluoropyrimidine prescribed as seen in Fig. 2: Early CRC regimen’s cycle cost for each claimed component as per the constructed treatment pathways and Fig. 3: Late CRC regimen’s Cycle cost for each claimed component as per the treatment pathways constructed - *(A – chemotherapy alone; B – Chemotherapy plus Bevacizumab; C – Chemotherapy plus Cetuximab; D – Single agents for refractory patients)* (FOLFOX: approx. €490 vs. €500 and FOLFIRI: approx. €420 vs. €460; CAPOX: approx. €660 for either and CAPIRI: approx. €590 vs. €610).

Moreover the cost difference between 5-FU and capecitabine monotherapy is less per cycle, regardless of stage, than the cost difference noted between the two treatments in the previously published study [25]. From Fig. 2: Early CRC regimen’s cycle cost for each claimed component as per the constructed treatment pathways and Fig. 3: Late CRC regimen’s Cycle cost for each claimed component as per the treatment pathways constructed - *(A – chemotherapy alone; B – Chemotherapy plus Bevacizumab; C – Chemotherapy plus Cetuximab; D – Single agents for refractory patients)* the difference in cost is less than €30 per cycle (5FU: approx. €290 vs. €300 and capecitabine: approx. €310 for early vs. late subgroups respectively) where the difference in the previously published study is 3 times more for 5-FU [25]. Therefore, based on cost, our results do not indicate a prescribing preference for the use of capecitabine, despite it’s proven oral availability, which is not consistent with previous research [45, 50–53]. This indicates multiple factors contribute to treatment decisions made by oncologists and patients. A literature review by Tariman and colleagues (2012) illustrated the complex nature of treatment decisions in older cancer patients. Apart from the many decision-making models that may be employed in the healthcare setting, factors including the oncologist’s medical expertise and practice type, a patient’s health related experience and perception of making a decision together with a patient’s family preference, burden and financial situation can all influence treatment choices [54].

Cost comparisons for newer therapies including bevacizumab and cetuximab could not be done, as these options are unavailable to patients in the South African public sector. However, treatment costs are increased substantially when a monoclonal antibody is added to conventional treatment, for example adding bevacizumab increases the cost per cycle by €811,31 and cetuximab by €1342,73. This result is in line with previous studies that show a lower cost of first- and second-line treatment with bevacizumab-containing regimens in comparison to cetuximab-containing regimens despite a similar efficacy [55–58]. This cost difference has shown to be more than $2000 per month per patient and alludes to a better value offering for funders [55–58].

The last notable comparison is the cost constituents for each regimen. Similarly to the previously published study, chemotherapy cost has a large contribution to overall cost per cycle regardless of the stage or regimen (Fig 2 Early CRC regimen’s cycle cost for each claimed component as per the constructed treatment pathways and Fig 3: Late CRC regimen’s Cycle cost for each claimed component as per the treatment pathways constructed - *(A – chemotherapy alone; B – Chemotherapy plus Bevacizumab; C – Chemotherapy plus Cetuximab; D – Single agents for refractory patients)*) [25]. However, administrative costs are a major cost driver in our cohort, which differs from the public sector cohort [25]. The administrative costs included a global and facility fee as set out by the medical scheme tariff. In comparison to the previously published study, the administrative costs are much higher in our cohort and do have a contributing effect on the total costs as seen in Fig 2: Early CRC regimen’s cycle cost for each claimed component as per the constructed treatment pathways and Fig 3: Late CRC regimen’s Cycle cost for each claimed component as per the treatment pathways constructed - *(A – chemotherapy alone; B – Chemotherapy plus Bevacizumab; C – Chemotherapy plus Cetuximab; D – Single agents for refractory patients)*. On average the cost contribution is between 10 and 45% of the total cost depending on the chemotherapy regimen. This is in line with previous research but is below the 70% threshold as found by Aitini and colleagues (2012) in their economic comparison of CAPOX and FOLFOX [59]. It is recommended that a time and motion study be undertaken in a similar manner to Herbst et al (2018) [25] so as to validate the tariffs charged and to allow for a more accurate comparison.

### Limitations

Due to the type of the claims captured on the claims database, the average cost per regimen doesn’t take into account for line of therapy but is the average for the stage of CRC diagnosed within the cohort. A comprehensive breakdown of the cost and equipment inclusions for the administration costs was unavailable therefore clarity and accuracy is lacking with respect to these costs and the total administration costs for intravenous regimens. This limits the comparison to the public sector as the administration costs calculated in Herbst et al’s 2018 cohort was extrapolated from a previous study that included all necessary equipment [25, 60]. The methodology utilized is based on the one previous study, other similar studies are lacking therefore this could not be validated against additional studies. Lastly, out-of-pocket (OOP) costs could not be determined using the claims database for this cohort as data only reflected the actual costs paid for by the funder. It would be beneficial to conduct a survey in line with previous research in order to quantify the OOP costs patients currently incur [61].

## Conclusions

This comparison highlights the vast differences in treatment access for the same disease within South Africa (4 public sector regimens vs. 12 private sector regimens) while providing insight into the cost and cost drivers of treatment in the South African healthcare sectors. The lack of comparable literature demonstrates the need for resource utilisation and outcome based studies in the country, which will ensure effective use of available resources so as to achieve equitable healthcare and value for treatment within the country, particularly as South Africa moves towards implementing universal health coverage through national health insurance.

## Supplementary information

**Additional file 1: Table S1.** Formuale used in calculations of treatment costs per patient.

## Data Availability

The datasets used and/or analysed during the current study are available from the corresponding author on reasonable request.

## Abbreviations

CRC: Colorectal Cancer
CAPOX: Capecitabine, oxaliplatin
CAPIRI: Capecitabine, irinotecan
FOLFOX: 5- Fluorouracil, oxaliplatin
FOLFIRI: 5- Fluorouracil, irinotecan
UMIC: Upper middle income country
NDoH: National Department of Health
EDP: Essential Drugs Program
EML: Essential Medicines List
STG: Standard Treatment Guidelines
HIC: High Income Countries
5-FU (+LV): 5- Fluorouracil, leucovorin (folinic acid)
CPI: Consumer Price Index
SEER: Surveillance, Epidemiology, and End Results Program
BMI: Body mass index
OOP: Out-of-pocket
