# Supplementary material for: Colorectal Cancer (CRC) treatment and associated costs in the public sector compared to the private sector in Johannesburg, South Africa
Source: BMC Health Serv Res. 2020 Apr 7;20:290. doi: 10.1186/s12913-020-05112-w (PMC7137465; doi:10.1186/s12913-020-05112-w)
Supplement: Supplementary file 1 — Additional file 1: Table S1. Formuale used in calculations of treatment costs per patient. [file 12913_2020_5112_MOESM1_ESM.docx]

Table S I Formuale used in calculations of treatment costs per patient

| $\boldsymbol{Average cost per claim for a medicine}= \frac{\sum total costs per medicine of the adjusted claim price}{Number of claims for the medicine}$ |
| --- |
| $\boldsymbol{A}\text{verage cost per regimen }\text{(cost component)}\text{=}\sum\text{ average cost per claim for all medicines within a chemotherapy regimen/*administration medicines/}\text{\#}\text{supportive medicines for a cycle}$  **administration medicines – solvents and diluting agents; carbohydrates; sodium chloride etc.*  *#supportive medicines – medicines for nausea and vomiting; corticosteroids; atropine if irinotecan-containing regimen* |
| $\text{Average cost per cycle for each regimen }\text{(cost component)}\text{ = }\frac{Average cost per regimen (cost component)}{\text{Average number of cycles per regimen (cost component)}}$ |
| $\boldsymbol{Total cost for regimen per cycle}= \sum average cost per cycle for each regimen \left( cost component \right)$  *= chemotherapy regimen cost + administration cost + supportive medicine cost + administrative cost* |
| $\boldsymbol{Total cost for x cycles}= Total cost for regimen per cycle X average number of cycles for that regimen$ |
